# The Impact of Sample Type on Vitamin D Quantification and Clinical Classification during Pregnancy

**DOI:** 10.3390/nu12123872

**Published:** 2020-12-18

**Authors:** Soriah M. Harvey, Vanessa E. Murphy, Peter G. Gibson, Michael Clarke, Megan E. Jensen

**Affiliations:** 1 Priority Research Centre Grow Up Well, Hunter Medical Research Institute, School of Medicine and Public Health, University of Newcastle, Callaghan, NSW 2305, Australia; soriah.harvey@uon.edu.au (S.M.H.); vanessa.murphy@newcastle.edu.au (V.E.M.); 2 Priority Research Centre for Healthy Lungs, University of Newcastle and Hunter Medical Research Institute, Callaghan, NSW 2305, Australia; peter.gibson@newcastle.edu.au; 3 Department of Respiratory and Sleep Medicine, John Hunter Hospital, New Lambton Heights, NSW 2305, Australia; 4 Metabolomics Australia, Centre for Microscopy, Characterisation and Analysis, The University of Western Australia, Perth, WA 6000, Australia; michael.clarke@uwa.edu.au; 5 School of Molecular Sciences, The University of Western Australia, Perth, WA 6000, Australia

**Keywords:** vitamin D, pregnancy, quantification, clinical, 25OHD, asthma, analytes, spectrophotometry, sample, plasma, serum, LC-MS/MS

## Abstract

Measurement of vitamin D status has significant use in clinical and research settings, including during pregnancy. We aimed to assess the agreement of total 25-hydroxyvitamin D (25(OH)D) concentration, and its three analytes (25-hydroxyvitamin D_3_ (25(OH)D_3_), 25-hydroxyvitamin D_2_ (25(OH)D_2_) and Epi-25-hydroxyvitamin D_3_ (Epi-25(OH)D_3_)), in plasma and serum samples collected during pregnancy, and to examine the proportion of women who change vitamin D status category based on sample type. Matching samples were collected from *n* = 114 non-fasting women between 12–25 weeks gestation in a clinical trial in Newcastle, Australia. Samples were analysed by liquid chromatography-tandem mass-spectrometry (LC-MS/MS) to quantify total 25(OH)D and its analytes and examined using Bland-Altman plots, Pearson correlation (r), intraclass correlation coefficient and Cohen’s Kappa test. Serum total 25(OH)D ranged from 33.8–169.8 nmol/L and plasma ranged from 28.6–211.2 nmol/L. There was a significant difference for total 25(OH)D based on sample type (measurement bias 7.63 nmol/L for serum vs plasma (95% Confidence Interval (CI) 5.36, 9.90, *p* ≤ 0.001). The mean difference between serum and plasma concentrations was statistically significant for 25(OH)D_3_ (7.38 nmol/L; 95% CI 5.28, 9.48, *p* ≤ 0.001) and Epi-25(OH)D_3_ (0.39 nmol/L; 95% CI 0.14, 0.64, *p* = 0.014). Of 114 participants, 28% were classified as vitamin D deficient (<50 nmol/L) or insufficient (<75 nmol/L) based on plasma sample and 36% based on serum sample. Nineteen (16.7%) participants changed vitamin D status category based on sample type. 25-hydroxyvitamin D quantification using LC-MS/MS methodology differed significantly between serum and plasma, yielding a higher value in plasma; this influenced vitamin D status based on accepted cut-points, which may have implications in clinical and research settings.

## 1. Introduction

The circulating concentration of 25-hydroxyvitamin D (25(OH)D) is considered the accepted clinical biomarker of vitamin D status [1]. With increasing awareness of the importance of vitamin D for maintaining optimal health by healthcare professionals, researchers and the public, requests for vitamin D quantification in human samples has increased in recent years [2,3], therefore ensuring the accuracy of analyses is highly relevant and important.

Testing is most commonly conducted through measurement of 25(OH)D via assay and quantification of 25(OH)D is routinely outsourced to be completed in external laboratories for clinical and research requirements. Previous studies have examined vitamin D in serum vs. plasma in small sample sizes, using assay techniques, and found serum and plasma to be mostly agreeable [4,5,6,7], but with evidence that 25(OH)D concentration may be higher in heparinised plasma compared to serum or ethylenediamine tetraacetic acid (EDTA) plasma [5]. However, this methodology has significant limitations to the usefulness and interpretation of the results, as assays have high result variability due to issues with standardisation, precision and accuracy [8]. Unlike immunoassays or high-pressure liquid chromatography (HPLC) methods, liquid chromatography tandem mass spectrophotometry (LC-MS/MS) offers higher specificity of detection, lower matrix interferences and high detectability of molecules present in low concentrations. This method is considered the gold standard method for vitamin D analysis, with the capability to measure 25(OH)D_2_, 25(OH)D_3_, and Epi-25(OH)D_3_ separately [9,10,11,12,13].

Four previous studies have compared sample type for 25(OH)D quantification using LC-MS/MS; however, the samples were collected from non-pregnant populations of small sample sizes. Zhang et al. (*n* = 25) and Abu Kassim et al. (*n* = 10) did not find a significant difference between EDTA plasma, heparin plasma and serum for 25(OH)D_2_ and 25(OH)D_3_ concentrations in healthy adults [14,15]. Differences between sample type for Epi-25(OH)D_3_ or total 25(OH)D were not examined. Albarhani et al. examined the usefulness of diluted plasma for quantification of 25(OH)D_3_ and Epi-25(OH)D_3_ compared to serum in umbilical cord blood samples (*n* = 20), and although their findings demonstrated close agreement for 25(OH)D_3_ in serum and plasma across two independent laboratories (r = 0.983) issues with analytical sensitivity in regards to limits of detection (LoD) for Epi-25(OH)D_3_ quantification highlighted issues with the use of diluted plasma instead of serum in other analytes of vitamin D [16]; 25(OH)D_2_ and total vitamin D were not reported. In a study of 13 healthy adults, Mena-Bravo et al. found that plasma and serum provided similar levels for 24,25(OH)D_3_, 25(OH)D_3_ and cholecalciferol (D_3_), while significantly higher concentrations of 1,25(OH)D_3_ were detected in plasma versus serum [17]. However, no study has compared quantification of vitamin D analytes in plasma and serum samples collected during pregnancy. Comparing these sample types is of benefit to the Vitamin D Standardisation Program (VDSP) that aims to promote 25(OH)D concentration measurements that are accurate (precise and true) and comparable over time, location and laboratory to improve clinical and public health practice world-wide [18].

It is noted that none of the previous studies examined comparability of total 25(OH)D serum and plasma concentrations, and no study has examined changes in clinical vitamin D status based on sample type during pregnancy. The effect of the sample type on resulting concentrations of 25(OH)D and its analytes during pregnancy needs to be identified clearly to ascertain the suitability of using serum and plasma interchangeably in clinical and research settings. Pregnancy is associated with various hormonal and physiological changes in the body, and with vitamin D also acting as a hormone, adaptive changes of vitamin D homeostasis and metabolite concentrations in pregnancy may have implications for the systemic circulation of total 25(OH)D and its analytes [19]. Vitamin D-Binding Protein levels increase drastically during pregnancy, and this can influence the concentration of free 25(OH)D as well as other analytes [20]. A 2019 study examined serum samples in pregnant (*n* = 88) and non-pregnant women (*n* = 20) and found differences in vitamin D metabolism across a range of analytes in pregnancy, as well as across gestation [21]. Whether there are important differences in vitamin D analyte concentrations, as well as clinical vitamin D status, based on sample type, is unknown.

The primary aim of this study was to investigate the comparability of vitamin D quantification using LC-MS/MS between serum and plasma samples collected during pregnancy in a large well-defined cohort of women enrolled in a clinical asthma trial. The secondary aim was to examine the proportion of women who change vitamin D category based on sample type.

## 2. Materials and Methods

Serum and plasma samples were collected from pregnant women who were enrolled in a clinical trial of asthma management during pregnancy, conducted in Newcastle, Australia. Details of the trial are previously described [22]. Briefly, women with current asthma, aged 18 years and older, were enrolled via antenatal clinics at the John Hunter Hospital in Newcastle, Australia. Written consent was obtained from participants before trial participation and ethics approval was granted by Hunter New England Health Human Research Ethics Committee (12/10/17/3.04, NSW HREC Reference No: HREC/12/HNE/357).

A non-fasting peripheral blood sample was collected by venepuncture (by a research nurse trained in phlebotomy) into a 6ml EDTA (1.8 mg/mL) plasma tube and 6mL plain serum tube at enrolment (between 12–25 weeks gestation), and processed within 60 min. All samples were centrifuged (3000 rpm) at 4 °C for ten minutes, aliquoted into Eppendorf tubes, and stored at −80 °C. All samples were collected between 2017–2019 and analysed in 2019. Samples were transported by courier on dry ice to the Centre for Microscopy, Characterisation & Analysis in Western Australia, for quantification of vitamin D via LC-MS/MS; this laboratory is certified by the Vitamin D Standardisation Program (VDSP) [18]. LC-MS/MS has been previously described [12]; briefly, samples were extracted using liquid-liquid extraction then separated using a 2D liquid chromatography UPLC system, followed by detection using tandem mass spectrometry. Total vitamin D was comprised of 25(OH)D_2_, 25(OH)D_3_ and Epi-25(OH)D_3_. The LoD for both 25(OH)D_3_ and Epi-25(OH)D_3_ was 2.0 nmol/L, and 3.0 nmol/L for 25(OH)D_2_. For blood samples with values below the LoD, a level equal to the detection limit was used, then divided by the square root of 2 (equal to 1.4 nmol/L for Epi-25(OH)D_3_ and 2.12 nmol/L for 25(OH)D_2_) [23]. Vitamin D sufficiency was defined as total 25OHD ≥75 nmol/L. Cut points for vitamin D insufficiency and deficiency were 50- <75 nmol/L and <50 nmol/L of total 25(OH)D, respectively [24].

### Statistical Analysis

The Shapiro Wilks test was used to determine normality of the data. Bland-Altman plots were used to compare the difference between plasma and serum concentrations of total 25(OH)D, 25(OH)D_2_, 25(OH)D_3_, epi-25(OH)D_3_ and detect proportional bias [25]. Pearson correlation (r) and intraclass correlation coefficient (ICC) was used to examine the agreement between the concentrations. The difference in mean values for total 25(OH)D, 25(OH)D_2_, 25(OH)D_3_ and Epi-25(OH)D_3_ were examined using the paired t-test with 95% confidence intervals (CI). A *p*-value < 0.05 was considered statistically significant. The mean, range, standard deviation (SD) and coefficient of variation (CV) were reported for each analyte by sample type. The proportion of participants that were vitamin D sufficient, insufficient and deficient were determined based on sample type, as well as any participants that changed category based on the use of plasma compared to serum for total 25(OH)D quantification. Cohen’s Kappa test was used to explore interrater reliability between participants vitamin D status and sample type. Statistics were computed with STATA IC v15.1 (StataCorp, College Station, TX, USA), and Microsoft Excel (v16.0.5083.1000, Microsoft Corporation, Santa Rosa, CA, USA).

## 3. Results

There were 114 matching serum and plasma samples. In total, 96.9% (221/228) samples were below the LoD for 25(OH)D_2_ and 11.8% (27/228) samples were below the LoD for Epi-25(OH)D_3_ (Table 1). The mean (±SD) for total 25(OH)D in serum was 86.77 ± 24.91 nmol/L (range 33.8–169.8 nmol/L). The mean for total 25(OH)D in plasma was 94.4 ± 28.8 nmol/L (range 28.6–211.2 nmol/L) (Table 1).

**Figure 1:**
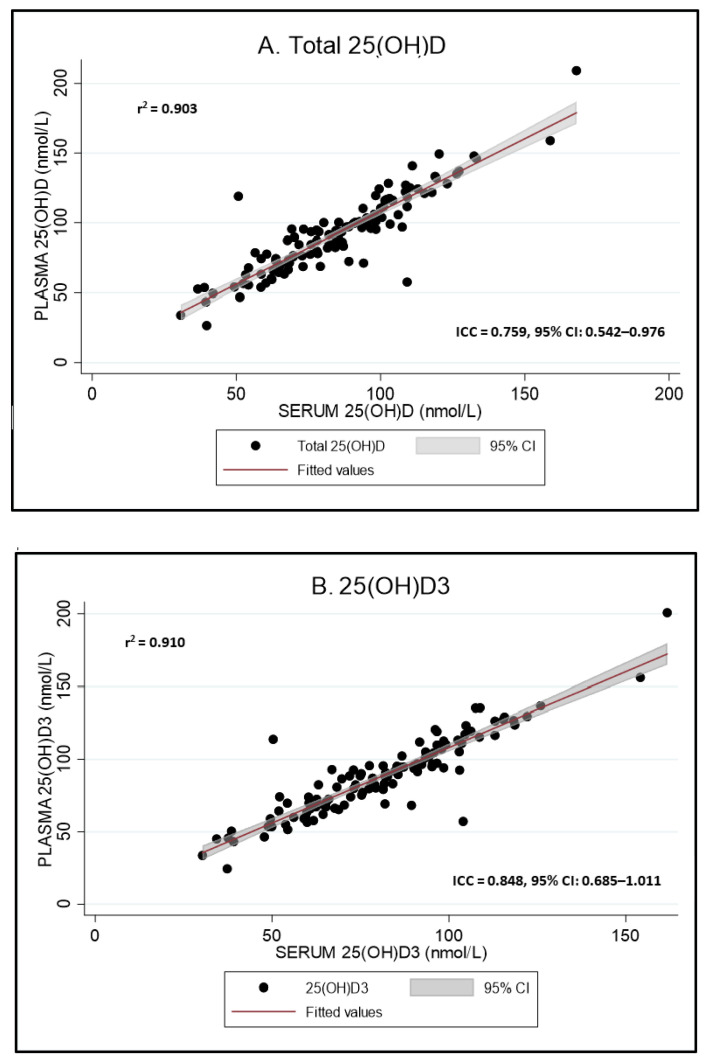

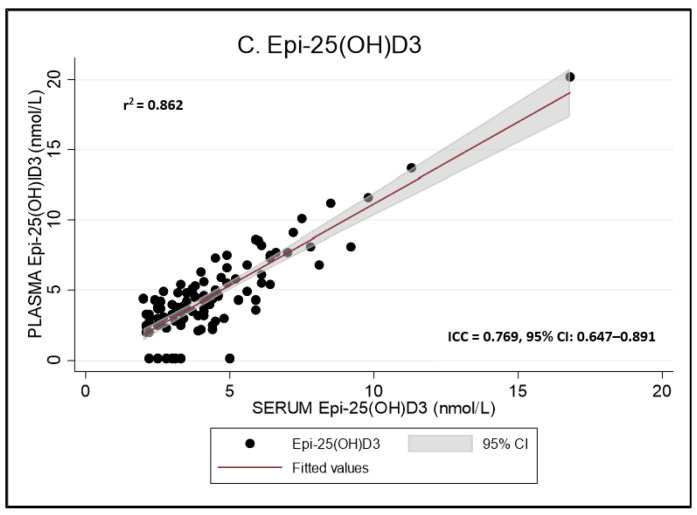
25(OH)D: 25-hydroxyvitamin D. (**A**–**C**) Scatter plot with Pearson (r^2^) and intraclass correlation coefficients (ICC) between serum and plasma concentrations of vitamin D analogues, quantified using LC-MS/MS. (**A**) Total 25(OH)D, (**B**) 25(OH)D_3_ and (**C**): Epi-25(OH)D_3_. 95% CI, 95% confidence interval. All results expressed as nmol/L.

Figure 1 illustrates the agreement between plasma and serum concentrations of total 25(OH)D (r^2^ = 0.903), 25(OH)D_3_ (r^2^ = 0.910) and Epi-25(OH)D_3_ (r^2^ = 0.862); correlation analyses were not conducted for 25OHD_2_ given the high percentage of samples below the LoD.

**Figure 2:**
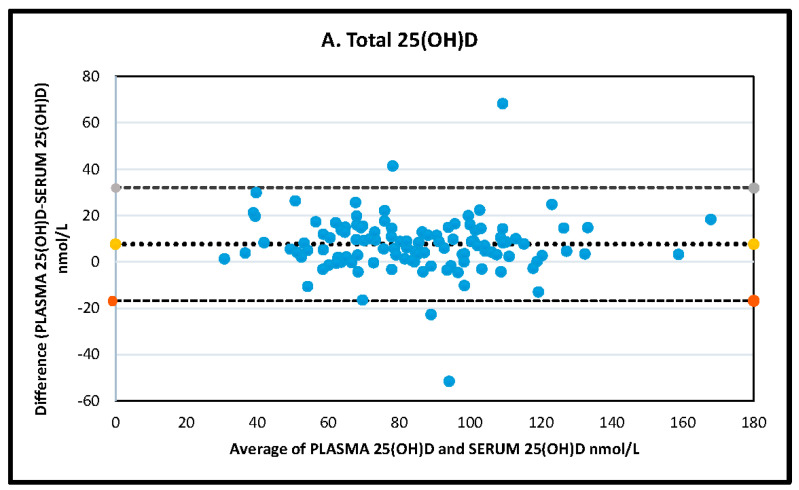

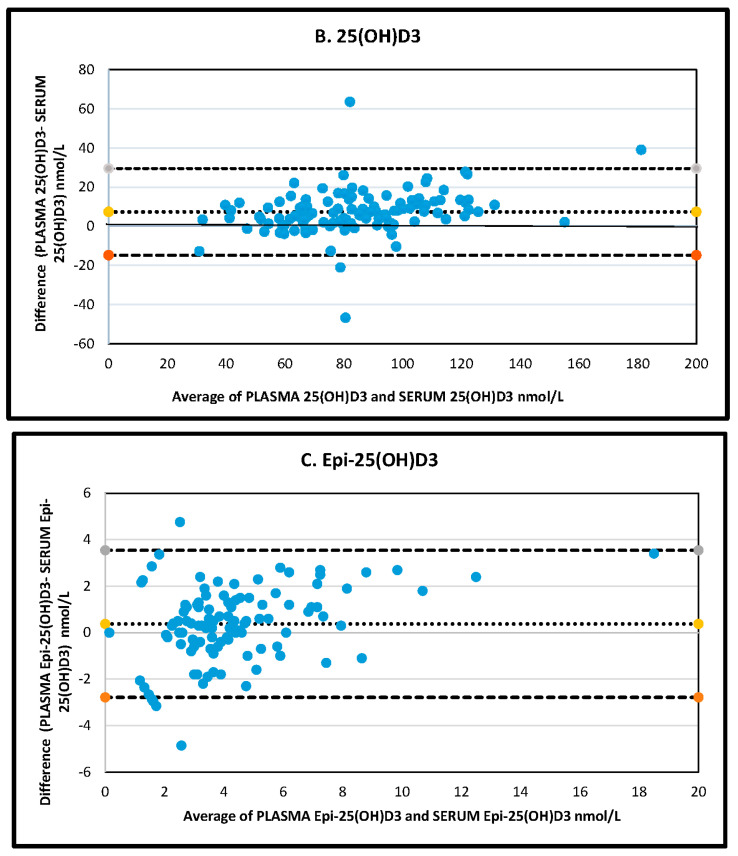
25(OH)D: 25-hydroxyvitamin D. (**A**–**C**) Bland-Altman Plot comparison between plasma and serum concentrations of vitamin D analytes quantified using liquid chromatography-tandem mass-spectrometry. (**A**) Total 25-hydroxyvitamin D (25(OH)D), (**B**) 25-hydroxyvitamin D_3_ (25(OH)D_3_) and (**C**) Epi-25-hydroxyvitamin D_3_ (25(OH)D_3_). Dotted line: mean difference (bias); dashed line: upper and lower 95% confidence intervals. All results expressed as nmol/L.

The Bland-Altman plot shows a mean total concentration bias for serum vs. plasma of 7.63 mol/L (95% CI 5.36, 9.90, *p* = < 0.001) for total 25(OH)D, 7.38 nmol/L (95% CI 5.28, 9.48 *p* = < 0.001) for 25(OH)D_3_, and 0.39 nmol/L (95% CI 0.14, 0.64, *p* = 0.003) for Epi-25(OH)D_3_ (Figure 2).

**Figure 3:**
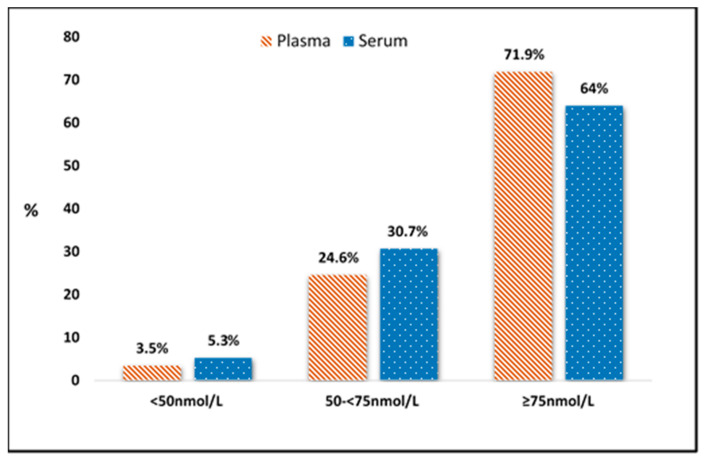
Vitamin D status category based on serum and plasma samples from women between 12–25 weeks gestation using LC-MS/MS.

In regards to vitamin D status based on sample type, for total 25(OH)D; results are shown in Figure 3 for serum and plasma. Cohen’s Kappa (κ) statistic showed moderate agreement between the participants’ vitamin D category based on serum compared to plasma (κ = 0.639, 95% CI 0.48, 0.80, *p* ≤ 0.001) with 83.3% agreement found. In total, 16.7% (*n* = 19) of participants changed vitamin D status category based on which sample type was used. Of the *n* = 19 that changed vitamin D status based on serum vs plasma samples; *n* = 12 participants changed from insufficient (<75 nmol/L) to sufficient (≥75 nmol/L), *n* = 3 changed from deficient (<50 nmol/L) to insufficient (50- <75 nmol/L), *n* = 1 changed from insufficient to deficient and *n* = 3 changed from sufficient to insufficient.

## 4. Discussion

This study is the first to examine the comparability of vitamin D quantification by LC-MS/MS in serum and plasma samples collected during pregnancy, and the first to examine the impact of sample type on vitamin D adequacy. Despite a relatively high Pearson and intraclass correlation between serum and plasma concentrations, examination of the concentration bias revealed a significant difference in the mean concentrations of total 25(OH)D in serum vs. plasma. Furthermore, we found 16.7% of participants changed category based on sample type, with 13% changing from deficient to insufficient or insufficient to sufficient with the use of plasma instead of serum.

These results are of concern in a clinical context, especially for pregnant women, as even small differences for patients close to cut-off points for vitamin D deficiency or insufficiency may result in misclassification of their vitamin D status, and may influence subsequent treatment decisions. The current national Australian pregnancy guidelines state that vitamin D supplementation may be considered for women with vitamin D levels <50 nmol/L, highlighting the impact this may have on recommended maternal supplementation, based on the vitamin D test result obtained [26]. Cut-points for vitamin D status and appropriate levels for optimal health have been highly controversial in recent years [27,28]. The Institute of Medicine recommends optimal levels of serum 25(OH)D to be >50 nmol/L based on requirements for bone health [29]. The Endocrine Society has opposed this as an adequate target level, and has identified a target 25(OH)D concentration for optimal health to be >75 nmol/L, based on a large body of evidence highlighting associated outcomes [27], with clinical guidelines recommending supplementation in children and adults to obtain this level [24].

Due to our large sample size, we were able to use Kappa statistic to explore participant’s vitamin D status category based on sample type [30,31]. Our resulting Kappa can be interpreted as moderate agreement between the participants resulting vitamin D status (deficient, insufficient or sufficient) based on serum or plasma sample type [31]. Whether a moderate level of agreement is adequate for the use of plasma and serum interchangeably has not been established, but our result does elucidate possible issues, as 13% of participants results could be categorised as false negatives for vitamin D insufficiency or deficiency when plasma was used instead of serum. A 2009 paper examined numerical specifications for trueness and analytical precision for routine analysis of serum/plasma 25(OH)D via immunoassay and LC-MS/MS for establishment of a reference measurement system [32]. Running several models for stringency and practical achievability; they found that, assuming a maximum tolerable limit of 20% clinical misclassifications, the quality goal for bias must be significantly less than 10% [32]. It is noted that the rate of 20% was chosen on an arbitrary basis and data are currently inadequate to ascertain a rate that could be considered acceptable to limit misclassification risk to the population [32]. Further research into acceptable agreement rates of vitamin D category based on sample type is warranted.

Assessing the comparability of sample types for results is important for future research and to assist in the VDSP’s goal of standardising vitamin D analysis. These results provide evidence of an important difference in vitamin D quantification, and assessment of vitamin D status, during pregnancy, based on sample type. These results are applicable to LC-MS/MS methods; whether there are differences with nonchromatographic methods based on radioimmunoassay’s, such as antibody assays, is unknown. Whether the results would be altered with different anticoagulants used is unknown and requires further investigation. Although serum and plasma are commonly used blood specimen types; they are not equivalent biological matrices [33]. Serum and plasma are used interchangeably for the quantification of vitamin D in some clinical and research settings, and this is the first study to supply evidence that there may be a significant difference in resulting vitamin D concentration using LC-MS/MS and status during pregnancy based on sample type. These results are applicable to pregnant populations; whether these differences in total 25(OH)D quantification by sample type, and subsequent categorisation of vitamin D status, are also seen in non-pregnant populations would require further research. Whilst evidence based recommendations are in place supporting the use of serum and plasma for other nutrients, such as vitamin A and E [16,34,35], similar recommendations are not in place for vitamin D. Serum is considered the appropriate sample type to use for vitamin D analysis and vitamin D reference ranges are based on serum levels, not plasma levels [24]. Our data further support this recommendation; however, where the use of plasma is unavoidable, development of a conversion factor would allow serum and plasma to be used interchangeably with more confidence in regards to the accuracy and precision of the results.

## Figures and Tables

**Table 1:** Mean, range, standard deviation and coefficient of variation for *n* = 114 matched serum and plasma samples collected from pregnant women.

Total 25(OH)D	Minimum (nmol/L)	Maximum (nmol/L)	Mean (nmol/L)	SD (nmol/L)	CV%	*p*-Value *
PLASMA	28.60	211.2	94.40	28.80	30.51	
SERUM	33.80	169.8	86.77	24.91	28.71	<0.001
25(OH)D_3_						
PLASMA	24.50	200.80	87.83	27.10	30.9	
SERUM	30.3	161.7	80.45	23.65	29.4	<0.001
25(OH)D_2_^‡^						
PLASMA	<3.0	6.3	2.23	0.54	24.22	
SERUM	<3.0	4.5	2.15	0.24	11.16	0.112
Epi-25(OH)D_3_^‡^						
PLASMA	<2.0	20.2	4.44	2.81	63.29	
SERUM	<2.0	16.8	4.05	2.28	56.30	0.003

* Paired *t*-test *p*-value comparing mean difference between serum and plasma for each analyte and total 25-hydroxyvitamin D (25(OH). Dusing liquid chromatography-tandem mass-spectrometry. Minimum, maximum, mean and SD are expressed in nmol/L. CV, coefficient of variation, expressed as percentage. SD, standard deviation. ^‡^: 96.9% of 25-hydroxyvitamin D_2_ (25(OH)D_2_) and 11.8% of Epi-25-hydroxyvitamin D_3_ (25(OH)D_3_) results were below the LoD’s and therefore imputed values were used.

## References

[1] National Institutes of Health (2014). Vitamin D: Fact Sheet for Health Professionals. https://ods.od.nih.gov/factsheets/VitaminD-HealthProfessional.

[2] Zhao S., Gardner K., Taylor W., Marks E., Goodson N. (2015). Vitamin D assessment in primary care: Changing patterns of testing. Lond. J. Prim. Care (Abingdon).

[3] Gordon L., Waterhouse M., Reid I.R., Neale R.E. (2020). The vitamin D testing rate is again rising, despite new MBS testing criteria. Med. J. Aust..

[4] Colak A., Toprak B., Dogan N., Ustuner F. (2013). Effect of sample type, centrifugation and storage conditions on vitamin D concentration. Biochem. Med..

[5] Yu C.-L., Falk R.T., Kimlin M.G., Rajaraman P., Sigurdson A.J., Horst R.L., Cosentino L.M., Linet M.S., Freedman D.M. (2010). The impact of delayed blood centrifuging, choice of collection tube, and type of assay on 25-hydroxyvitamin D concentrations. Cancer Causes Control CCC.

[6] Lissner D., Mason R.S., Posen S. (1981). Stability of vitamin D metabolites in human blood serum and plasma. Clin. Chem..

[7] Norris R.L., Thomas M.J., Craswell P.W. (1986). Assessment of a two-step high-performance liquid chromatographic assay using dual-wavelength ultraviolet monitoring for 25-hydroxyergocalciferol and 25-hydroxycholecalciferol in human serum or plasma. J. Chromatogr..

[8] Farrell C.-J.L., Martin S., McWhinney B., Straub I., Williams P., Herrmann M. (2012). State-of-the-Art Vitamin D Assays: A Comparison of Automated Immunoassays with Liquid Chromatography-Tandem Mass Spectrometry Methods. Clin. Chem..

[9] Yetley E.A., Pfeiffer C.M., Schleicher R.L., Phinney K.W., Lacher D.A., Christakos S., Eckfeldt J.H., Fleet J.C., Howard G., Hoofnagle A.N. (2010). NHANES monitoring of serum 25-hydroxyvitamin D: A roundtable summary. J. Nutr..

[10] de la Hunty A., Wallace A.M., Gibson S., Viljakainen H., Lamberg-Allardt C., Ashwell M. (2010). UK Food Standards Agency Workshop Consensus Report: The choice of method for measuring 25-hydroxyvitamin D to estimate vitamin D status for the UK National Diet and Nutrition Survey. Br. J. Nutr..

[11] Chen H., McCoy L., Schleicher R., Pfeiffer C. (2008). Measurement of 25-hydroxyvitamin D-3 (25OHD(3)) and 25-hydroxyvitamin D-2 (25OHD(2)) in human serum using liquid chromatography-tandem mass spectrometry and its comparison to a radioimmunoassay method. Clin. Chim. Acta Int. J. Clin. Chem..

[12] Clarke M.W., Tuckey R.C., Gorman S., Holt B., Hart P.H. (2013). Optimized 25-hydroxyvitamin D analysis using liquid-liquid extraction with 2D separation with LC/MS/MS detection, provides superior precision compared to conventional assays. Metabolomics.

[13] Wise S.A., Phinney K.W., Tai S.S., Camara J.E., Myers G.L., Durazo-Arvizu R., Tian L., Hoofnagle A.N., Bachmann L.M., Young I.S. (2017). Baseline Assessment of 25-Hydroxyvitamin D Assay Performance: A Vitamin D Standardization Program (VDSP) Interlaboratory Comparison Study. J. AOAC Int..

[14] Zhang S.W., Jian W., Sullivan S., Sankaran B., Edom R.W., Weng N., Sharkey D. (2014). Development and validation of an LC-MS/MS based method for quantification of 25 hydroxyvitamin D2 and 25 hydroxyvitamin D3 in human serum and plasma. J. Chromatogr. B Anal. Technol. Biomed. Life Sci..

[15] Abu Kassim N.S., Gomes F.P., Shaw P.N., Hewavitharana A.K. (2016). Simultaneous quantitative analysis of nine vitamin D compounds in human blood using LC-MS/MS. Bioanalysis.

[16] Albarhani A.A., Collier F., Greaves R.F., Ponsonby A.L., Allen K.J., Vuillermin P.J., Roche P., Clarke M.W., BIS Steering Committee (2015). Vitamins D and A can be successfully measured by LC-MS/MS in cord blood diluted plasma. Clin. Biochem..

[17] Mena-Bravo A., Priego-Capote F., Luque de Castro M.D. (2015). Study of blood collection and sample preparation for analysis of vitamin D and its metabolites by liquid chromatography-tandem mass spectrometry. Anal. Chim. Acta.

[18] Sempos C.T., Vesper H.W., Phinney K.W., Thienpont L.M., Coates P.M. (2012). Vitamin D status as an international issue: National surveys and the problem of standardization. Scand. J. Clin. Lab. Investig. Suppl..

[19] Karras S.N., Wagner C.L., Castracane V.D. (2018). Understanding vitamin D metabolism in pregnancy: From physiology to pathophysiology and clinical outcomes. Metabolism.

[20] Fernando M., Ellery S.J., Marquina C., Lim S., Naderpoor N., Mousa A. (2020). Vitamin D-Binding Protein in Pregnancy and Reproductive Health. Nutrients.

[21] Beentjes C.H.L., Taylor-King J.P., Bayani A., Davis C.N., Dunster J.L., Jabbari S., Mirams G., Jenkinson C., Kilby M., Hewison M. (2019). Defining vitamin D status using multi-metabolite mathematical modelling: A pregnancy perspective. J. Steroid Biochem. Mol. Biol..

[22] Murphy V.E., Jensen M.E., Mattes J., Hensley M.J., Giles W.B., Peek M.J., Bisits A., Callaway L.K., McCaffery K., Barrett H.L. (2016). The Breathing for Life Trial: A randomised controlled trial of fractional exhaled nitric oxide (FENO)-based management of asthma during pregnancy and its impact on perinatal outcomes and infant and childhood respiratory health. BMC Pregnancy Childbirth.

[23] Bartolucci A.A. (2016). Limits of Calibration. Introduction to Statistical Analysis of Laboratory Data.

[24] Holick M.F., Binkley N.C., Bischoff-Ferrari H.A., Gordon C.M., Hanley D.A., Heaney R.P., Hassan Murad M., Weaver C.M. (2011). Evaluation, Treatment, and Prevention of Vitamin D Deficiency: An Endocrine Society Clinical Practice Guideline. J. Clin. Endocrinol. Metab..

[25] Bland J.M., Altman D.G. (1986). Statistical methods for assessing agreement between two methods of clinical measurement. Lancet.

[26] Department of Health (2018). Clinical Practice Guidelines: Pregnancy Care.

[27] Vieth R., Holick M.F., Feldman D. (2018). The IOM—Endocrine Society Controversy on Recommended Vitamin D Targets. Support of the Endocrine Society Position.

[28] Bouillon R., Rosen C., Feldman D. (2018). The IOM—Endocrine Society Controversy on Recommended Vitamin D Targets. Support of the IOM Position.

[29] Del Valle H.B., Yaktine A.L., Taylor C.L., Ross A.C. (2011). Dietary Reference Intakes for Calcium and Vitamin D.

[30] Sim J., Wright C.C. (2005). The Kappa Statistic in Reliability Studies: Use, Interpretation, and Sample Size Requirements. Phys. Ther..

[31] McHugh M.L. (2012). Interrater reliability: The kappa statistic. Biochem. Med..

[32] Stöckl D., Sluss P.M., Thienpont L.M. (2009). Specifications for trueness and precision of a reference measurement system for serum/plasma 25-hydroxyvitamin D analysis. Clin. Chim. Acta.

[33] Sapan C.V., Lundblad R.L. (2006). Considerations regarding the use of blood samples in the proteomic identification of biomarkers for cancer diagnosis. Cancer Genom. Proteom..

[34] Castle M.C., Cooke W.J. (1985). Measurement of vitamin E in serum and plasma by high performance liquid chromatography with electrochemical detection. Ther. Drug Monit..

[35] Greaves R.F., Woollard G.A., Hoad K.E., Walmsley T.A., Johnson L.A., Briscoe S., Koetsier S., Harrower T., Gill J.P. (2014). Laboratory medicine best practice guideline: Vitamins a, e and the carotenoids in blood. Clin. Biochem. Rev..

